# Continuous Production of Human Epidermal Growth Factor Using *Escherichia coli* Biofilm

**DOI:** 10.3389/fmicb.2022.855059

**Published:** 2022-04-12

**Authors:** Mengting Li, Zhenyu Wang, Miao Zhou, Chong Zhang, Kaiqi Zhi, Shuli Liu, Xiujuan Sun, Zhi Wang, Jinle Liu, Dong Liu

**Affiliations:** ^1^State Key Laboratory of Materials-Oriented Chemical Engineering, College of Biotechnology and Pharmaceutical Engineering, Nanjing Tech University, Nanjing, China; ^2^School of Life Sciences, Zhengzhou University, Zhengzhou, China; ^3^School of Chemical Engineering and Energy, Zhengzhou University, Zhengzhou, China; ^4^Institute of Industrial Biotechnology, Jiangsu Industrial Technology Research Institute (JITRI), Nanjing, China

**Keywords:** *Escherichia coli*, biofilm formation, hEGF secretion, immobilized fermentation, biofilm-based fermentation

## Abstract

Increasing demand for recombinant proteins necessitates efficient protein production processes. In this study, a continuous process for human epidermal growth factor (hEGF) secretion by *Escherichia coli* was developed by taking advantage of biofilm formation. Genes *bcsB*, *fimH*, and *csgAcsgB* that have proved to facilitate biofilm formation and some genes *moaE*, *yceA*, *ychJ*, and *gshB* potentially involved in biofilm formation were examined for their effects on hEGF secretion as well as biofilm formation. Finally, biofilm-based fermentation processes were established, which demonstrated the feasibility of continuous production of hEGF with improved efficiency. The best result was obtained from *ychJ*-disruption that showed a 28% increase in hEGF secretion over the BL21(DE3) wild strain, from 24 to 32 mg/L. Overexpression of *bcsB* also showed great potential in continuous immobilized fermentation. Overall, the biofilm engineering here represents an effective strategy to improve hEGF production and can be adapted to produce more recombinant proteins in future.

## Introduction

With increasing demand for pharmaceutical proteins nowadays, more and more people are devoted to development of efficient biomanufacturing processes for proteins. *Escherichia coli* (*E. coli*), as one of the most commonly used host bacteria to express recombinant proteins, has many advantages. For example, high cell density cultures are easily achieved (Lee, 1996), transformation with exogenous genes is fast (Pope and Kent, 1996), and its use as a cell factory is well-established (Rosano and Ceccarelli, 2014). However, commercial production of recombinant proteins by *E. coli* has remained a challenge. One of the most important problems is that *E. coli* has a poor ability to secrete proteins, which causes formation of inclusion bodies, low production titers, and complicated downstream purification procedures (Kim et al., 2015). To improve protein secretion efficiency in *E. coli*, some common strategies have been developed, such as selection of efficient signal peptides like the signal sequences from OmpA (Sun et al., 2019) and PelB (Mohajeri et al., 2016) that usually target proteins to the secretion machinery efficiently. Modification of key transporter proteins or auxiliary proteins involved in transmembrane transport also has been applied to increase the efficiency of protein secretion (Low et al., 2010). In addition, fusion of some peptide tags or proteins, such as glutathione S-transferase (GST), also have proved to increase protein expression and extracellular secretion. Other strategies to improve protein secretion include fermentation process parameters control and medium optimization, such as addition of surfactants, osmolytes, metal ions, and glycine to the medium.

Recombinant human epidermal growth factor (rhEGF), a potential therapeutic protein that has been widely used as a healing agent for various chronic wounds, was successfully produced extracellularly in *E. coli* BL21(DE3) using pectate lyase B (PelB) signal peptide (Sriwidodo et al., 2019). Secretion of hEGF into the medium greatly simplifies product recovery, since no cell disruption is required, and it avoids intracellular proteolysis by periplasmic proteases (Choi and Lee, 2004). However, hEGF fermentation was operated in a free-cell batch fermentation mode, facing the challenge of decreased cell viability by stress conditions such as shear forces during fermentation, wherein cells cannot be reused and increase the production cost. In order to alleviate the bottleneck, immobilized fermentation taking advantages of biofilm formation has been proposed as an alternative to traditional free-cell batch fermentation. Biofilm is known as complex cell communities adhering onto biological or abiotic surfaces. It is a renewable living system. Biofilm has special superiorities such as protection of cells by biofilm matrix, enhanced metabolic activities, and repeated use of cells, which can significantly increase cell tolerance and shorten fermentation time (Zhao et al., 2015). An immobilized fermentation system for small-molecule chemicals such as shikimate (Ding et al., 2021) and L-threonine (Chen et al., 2019) has been developed by taking advantages of biofilm formation. However, so far there have been few studies on application of biofilm to the production of recombinant protein, probably because protein secretion is more complex. Integration of biofilm-based fermentation with protein secretion would enable continuous production of a protein, which is of great importance to improve productivities and reduce fermentation cost.

Biofilm formation is a complex process, involving coordinating a series of changes in physiological processes and cell surface structures (Sauer, 2003). For the biofilm formation in *E. coli*, external structures play a very important role in biofilm extracellular matrix components formation. Type I fimbriae, encoded by the *fim* genes, play a necessary role in attaching to inanimate surfaces initially (Nishiyama et al., 2008). Curli fimbriae, encoded by the *csg* genes, is indispensable for enhancing the cell-to-surface interaction and facilitating the cell-to-cell communication (Cookson et al., 2002). Cellulose, produced by the *bcs* genes, is one of the most important architectural elements in spatially structured biofilms (Zogaj et al., 2001). These extracellular bacterial structures work together to stabilize the biofilm matrix and form an intricate three-dimensional structure. Cells encased in biofilms could use these structures to obtain nutrients and withstand unfavorable conditions, which is a desired characteristic during fermentation (Prigent-Combaret et al., 1999).

**FIGURE 1:**
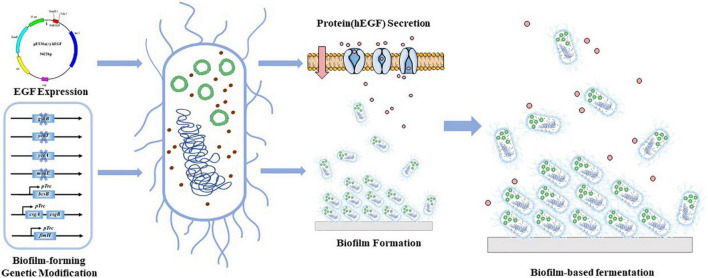
A graphical illustration of biofilm-based secretion of hEGF in this study.

In this study, we aimed to develop a biofilm-based fermentation process for continuous production of hEGF in *E. coli* (Figure 1). Genes that have often been shown to facilitate biofilm formation (Type I fimbriae formation gene *fimH*, cellulose formation gene *bcsB*, and curli fimbriae formation genes *csgA* and *csgB*) were manipulated to enhance biofilm formation. Besides, some new genes potentially involved in biofilm formation in *E. coli* according to a previous study (Kritikos et al., 2017) were also investigated, including a molybdopterin synthase gene *moaE*, a glutathione synthase gene *gshB*, a tRNA U34 hydroxylase gene *yceA*, and an unknown protein gene *ychJ*. At the same time, manipulations of these genes were evaluated for their effects on protein secretion. Finally, biofilm-based hEGF secretion systems were established, which demonstrated the feasibility of continuous production of hEGF with improved efficiency.

## Materials and Methods

### Strains and Plasmids

*Escherichia coli* MG1655 and BL21(DE3) were used in this study.

All strains and plasmids used in this work are listed in Table 1. Primers used in this study are listed in Supplementary Table 1. Vector pET30a-hEGF was used to express hEGF in BL21(DE3) and its modified strains. The biofilm genes *bcsB*, *fimH*, and *csgAcsgB* were amplified from the genomic DNA of *E. coli* MG1655. These genes were individually ligated to the expression plasmid pBbE1a (with restriction enzyme *Bgl*II and *Avr*II) by using the ClonExpress II One Step Cloning Kit C112-01 (Vazyme, Nanjing, China) and then were expressed in MG1655 and BL21(DE3), respectively, resulting in plasmid-based expression strains MG1655-*bcsB*^+^, MG1655-*csgAcsgB*^+^, MG1655-*fimH*^+^, BL21-*bcsB*^+^, BL21-*csgAcsgB*^+^, and BL21-*fimH*^+^, respectively. For genome-integrated expression, the *bcsB*, *fimH*, and *csgAcsgB* were inserted in the *moaE* site by CRISPR, under the *trc* promoter, resulting in genome-integrated expression strains MG1655-bcsB*, MG1655 -csgAcsgB*, MG1655-fimH*, BL21-bcsB*, BL21-csgAcsgB*, and BL21-fimH*. The genes *moaE*, *gshB*, *yceA*, and *ychJ* in *E. coli* MG1655 and BL21(DE3) were individually deleted by CRISPR, using pCas and pTarget (Jiang et al., 2015), resulting in MG1655 Δ*moaE*, MG1655 Δ*gshB*, MG1655 Δ*yceA*, MG1655 Δ*ychJ*, BL21 Δ*moaE*, BL21 Δ*gshB*, BL21 Δ*yceA*, and BL21 Δ*ychJ*.

**TABLE 1:** Strains and plasmids used in this study.

Strains or plasmids	Relevant characteristics	Sources
Strains		
*E. coli* MG1655		Stored in our lab
*E. coli* BL21(DE3)		Stored in our lab
*E. coli* MG1655-*bcsB*^+^	Plasmid-based expression of *bcsB*	This study
*E. coli* BL21(DE3)-*bcsB*^+^	Plasmid-based expression of *bcsB*	This study
*E. coli* MG1655-*csgAcsgB*^+^	Plasmid-based expression of *csgAcsgB*	This study
*E. coli* BL21(DE3)-*csgAcsgB*^+^	Plasmid-based expression of *csgAcsgB*	This study
*E. coli* MG1655-*fimH*^+^	Plasmid-based expression of *fimH*	This study
*E. coli* BL21(DE3)-*fimH*^+^	Plasmid-based expression of *fimH*	This study
*E. coli* MG1655-bcsB*	Genome-integrated expression of *bcsB*	This study
*E. coli* BL21(DE3)-bcsB*	Genome-integrated expression of *bcsB*	This study
*E. coli* MG1655-csgAcsgB*	Genome-integrated expression of *csgAcsgB*	This study
*E. coli* BL21(DE3)-csgAcsgB*	Genome-integrated expression of *csgAcsgB*	This study
*E. coli* MG1655-fimH*	Genome-integrated expression of *fimH*	This study
*E. coli* BL21(DE3)-fimH*	Genome-integrated expression of *fimH*	This study
*E. coli* MG1655 Δ*moaE*	Disruption of *moaE*	This study
*E. coli* BL21(DE3) Δ*moaE*	Disruption of *moaE*	This study
*E. coli* MG1655 Δ*gshB*	Disruption of *gshB*	This study
*E. coli* BL21(DE3) Δ*gshB*	Disruption of *gshB*	This study
*E. coli* MG1655 Δ*yceA*	Disruption of *yceA*	This study
*E. coli* BL21(DE3) Δ*yceA*	Disruption of *yceA*	This study
*E. coli* MG1655 Δ*ychJ*	Disruption of *ychJ*	This study
*E. coli* BL21(DE3) Δ*ychJ*	Disruption of *ychJ*	This study
Plasmids		
pCas	CRISPR-Cas editing	Prof. Sheng Yang
pTarget	CRISPR-Cas editing	Prof. Sheng Yang
pBbE1a		Stored in our lab
pBbE1a-*bcsB*^+^	Plasmid-based expression of *bcsB*	This study
pBbE1a-*csgAcsgB*^+^	Plasmid-based expression of *csgAcsgB*	This study
pBbE1a-*fimH*^+^	Plasmid-based expression of *fimH*	This study
pET30a-hEGF	hEGF expression	Stored in our lab

### Media and Growth Conditions

All recombinant strains were routinely cultured at 37°C and 200 rpm in LB medium, which contained 5 g/L yeast extract, 10 g/L tryptone, and 10 g/L NaCl. Solid media were prepared in all cases by adding 1.5% (w/v) agar. When necessary, 6 g/L glucose was added into LB medium (hereafter named LBG medium) to evaluate biofilm formation. For genome-integrated expression, *E. coli* strains contained only a pET30a-hEGF plasmid and were cultured with 50 μg/ml kanamycin. For plasmid-based expression, *E. coli* strains were cultured with 50 μg/ml kanamycin and 100 μg/ml ampicillin at 25°C after IPTG induction.

### Characterization of Biofilm Formation in 96-Well Plates

Because the basic dye crystal violet binds to negatively charged surface molecules and polysaccharides in the biofilm matrix, biofilm biomass quantification was measured by crystal violet staining in 96-well polystyrene plates (Li et al., 2003). Each well was filled with 200 μl working volume, and IPTG was added as necessary. Test strains were grown at 25^°^C under static conditions without shaking. After cultivation, LB or LBG liquid medium was poured out, wells were washed with phosphate buffer for two to three times to remove loosely adherent cells, and then fixed with methanol at 4°C for 15 min. Bacteria attached to the bottom of plates were then stained with 200 μl of 1% crystal violet for 15 min, after which unbound crystal violet was washed by water gently. Subsequently, cell-bound crystal violet was released from the bacterial cells by addition of 200 μl of 33% glacial acetic acid. After slight shaking at room temperature for 30 min, the concentrations of crystal violet in solution were measured as absorbance at 570 nm and used to represent the relative amount of biofilm biomass.

### Congo Red Assay

Congo red (CR) has the ability to bind with cellulose and amyloid fibers and thus was used to stain biofilm cells (Jiang et al., 2018). Seed cultures were inoculated and grown in LB medium (with 100 μg/ml ampicillin as necessary) for 12 h at 37°C. Experimental cultures were grown at 25°C, 150 rpm and were induced with 0.5 mM IPTG for 20 h. Subsequently, the cells were harvested from the culture broth by centrifugation. The collected cell pellets were washed with phosphate-buffered saline (PBS, pH 7.4) twice and then resuspended in PBS to an OD_600_ of 6.0. An aliquot of 10 μl CR solution (10 mg/ml) was added to 1 mL of cell suspension, or 1 ml of PBS as control. After 10 min, the supernatants and bacteria cells were separated by centrifugation. Residual amount of CR in the supernatant was measured as absorbance at 485 nm. The amount of bound CR was determined by subtracting the supernatant absorbance from the absorbance of control.

### Fermentation for Human Epidermal Growth Factor Production

For free-cell fermentation, a single colony from the solid plate was inoculated into 5 ml liquid LB medium at 37°C and 220 rpm for 12 h to obtain seed culture. Then, the seed culture was transferred to 50 ml LB medium in 250-ml flasks containing 50 μg/ml kanamycin (and 100 μg/ml ampicillin for strains contained biofilm gene expressing plasmids) with 1% (v/v) inoculum and grown at 37°C to OD_600_ of 0.6–0.8. The cells were then induced with 1.0 mM IPTG for 48 h and the cultures were pelleted by centrifugation at 8,000 rpm for 3 min. The supernatants were collected for subsequent analysis of hEGF production.

For biofilm-based continuous fermentation, 40 g/L of cotton towel (ZIPUTAO, Zhengzhou, China) were added into the fermentation medium as carrier for biofilm formation. The cotton towel was cut to pieces of size of 10 cm × 10 cm and then they were sterilized at 121^°^C for 20 min within the fermentation medium. At the end of the first batch, the fermented broth was removed from the flask but the carrier covered by biofilm was left for the second batch. After adding fresh culture medium, the second batch was initiated under the same conditions described above until the hEGF titer was stable. The subsequent batches were operated in the same way as above.

### Analytical Method for Human Epidermal Growth Factor Production

Since hEGF was secreted into the extracellular medium in this study, the supernatant was collected by centrifugation. The secreted hEGF in the supernatant was detected by sodium dodecyl sulfate-polyacrylamide gel electrophoresis (SDS-PAGE), and the production was estimated by quantitative analysis using Image Lab version 5.2.1. Supernatant of 21 μl was mixed with 7 μl of SDS-PAGE loading buffer and the sample was then boiled at 95°C for 10 min. Tricine-SDS-PAGE Gel Preparation Kit (Sangon Biotech) was used in this study to prepare gels (16.5%) for electrophoresis. The total protein content was measured by the Bradford method (Bradford, 1976) as a reference.

## Results and Discussion

### Genetic Modification of Biofilm Formation

Besides the *bcsB*, *fimH*, and *csgAcsgB* which are well-known favorable genes for biofilm formation in *E. coli*, four potential genes were also examined. The *moaE* encodes a protein involved in molybdopterin biosynthesis. A previous study showed that failure to synthesize molybdopterin led to elevated levels of the ubiquitous second messenger c-di-GMP, and thus more biofilm (Hengge, 2009). The gene *gshB* encodes a glutathione synthetase. Glutathione has many roles within the cell such as clearing free radicals and enhancing resistance to some antibiotics (He et al., 2008). It was demonstrated that glutathione alone was sufficient to disrupt immature and mature biofilms in *Pseudomonas aeruginosa* (Klare et al., 2016). However, it is not clear how *yceA* that encodes a tRNA U34 hydroxylase and *ychJ* of unknown function act on biofilm. According to a previous study, when these genes were individually disrupted, the cells bound to CR obviously and turned red on the agar plate, suggesting that these genes might have certain effects on biofilm formation (Kritikos et al., 2017). Therefore, overexpressing *bcsB*, *csgAcsgB*, and *fimH*, and disrupting *moaE*, *gshB*, *yceA*, and *ychJ* were carried out in this study.

**FIGURE 2:**
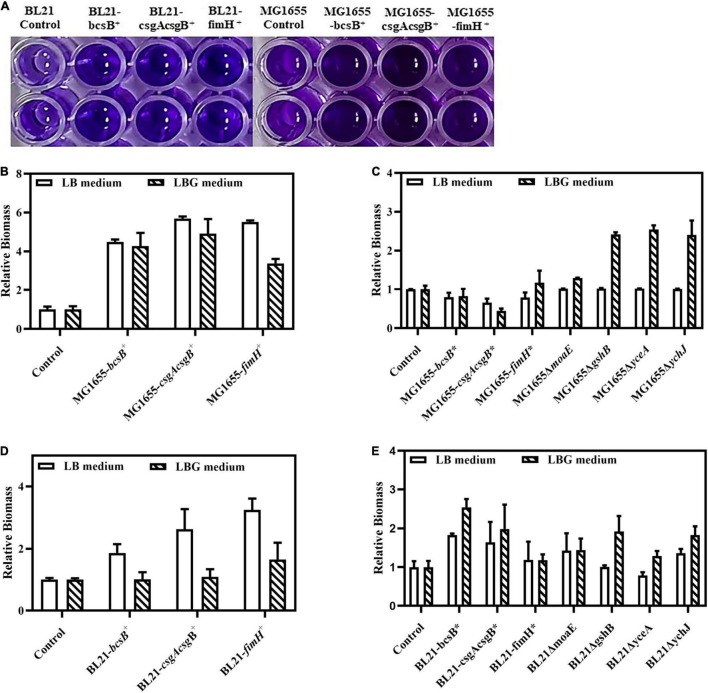
Crystal violet staining based quantification of biofilm biomass. **(A)** Crystal violet staining of biofilm in 96-well plates. **(B)** Relative biofilm biomass of MG1655 strains with plasmid-based overexpression of biofilm related genes. **(C)** Relative biofilm biomass of MG1655 strains with genome-integrated expression of or disruption of biofilm related genes. **(D)** Relative biofilm biomass of BL21 strains with plasmid-based overexpression of biofilm related genes. **(E)** Relative biofilm biomass of BL21 strains with genome-integrated expression of or disruption of biofilm related genes. The biofilm amounts of control strains were set as 1.0 and those of the corresponding recombinant strains were expressed as fold changes.

**FIGURE 3:**
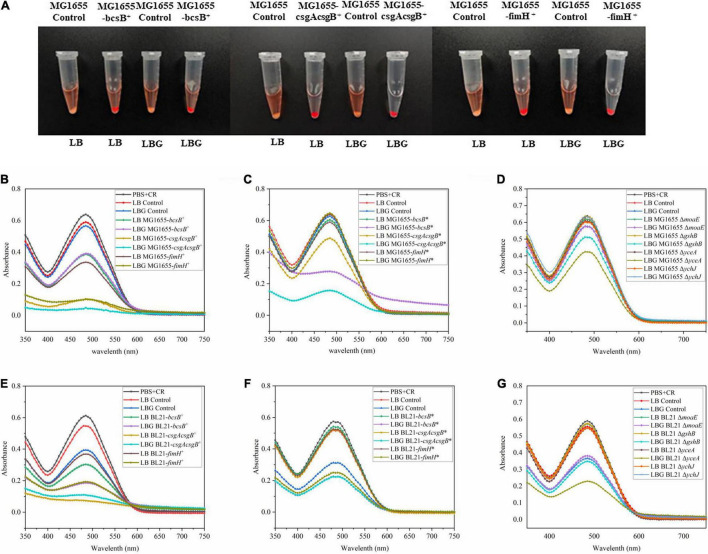
CR binding assay for certain biofilm matrix components. **(A)** CR adsorption by MG1655 cells overexpressing biofilm related genes grown in LB or LBG medium. **(B–G)** Residual amount of CR in the supernatant after adsorption with different recombinants cells, measured as absorbance at 485 nm [see section “Congo Red (CR) Assay” for experimental details].

The *bcsB*, *csgAcsgB*, and *fimH* genes were first overexpressed by a plasmid. Results showed that, in both *E. coli* BL21(DE3) and MG1655 strains, these genes could apparently promote biofilm formation. When the biofilm in the 96-well plate was stained with crystal violet, the color of these recombinant strains was much darker than that of wild strain (Figure 2A). The biofilm mass quantified through the crystal violet staining was 1.8- to 3.3-fold higher than that of the control strain BL21(DE3), or 4.8- to 5.7-fold higher than the that of the control MG1655. Adsorption of CR by the biofilm was also studied to evaluate biofilm matrix components like cellulose and amyloid fibers. Results showed that adsorption of CR increased greatly upon expression of *bcsB*, *csgAcsgB*, or *fimH*, as indicated by the much redder cell pellets and clearer supernatant (Figure 3A). When LB medium was supplemented with glucose (that is LBG medium), adsorption of CR was more obvious, although this seemed to decrease biofilm mass to some extent.

Since overexpression of *bcsB*, *csgAcsgB*, or *fimH* gene could effectively enhance biofilm formation, they were subsequently integrated into the *E. coli* genome under the *trc* promoter individually to obtain more stable industrial strains. Results showed that although genome-integrated expression of the genes was not as effective as plasmid-based overexpression probably due to a lower copy number, they could still promote biofilm formation in BL21(DE3). When grown in LB medium and induced with IPTG, the biofilm mass of the integrated strains BL21-bcsB* and BL21-csgAcsgB* was 1.6–1.8-fold higher than the control (Figure 2E). Adsorption of CR by the integrated strains MG1655-bcsB* and MG1655-*csgAcsg*B* in LBG medium was also more obvious (Figure 3C). In some cases, *E. coli* BL21(DE3) and MG1655 differed in biofilm formation. It is known that BL21(DE3) is defective in cellulose biosynthesis and has no flagella due to a 41 kb IS1-mediated deletion of the flagella genes (Jeong et al., 2009). Also, BL21(DE3) is not able to respond to autoinducer-2 because the *lsr* quorum sensing gene cluster is removed (Studier et al., 2009). These phenotypes are closely related to biofilm formation. Therefore, it was understandable that trends were sometimes different between the two *E. coli* strains.

**FIGURE 4:**
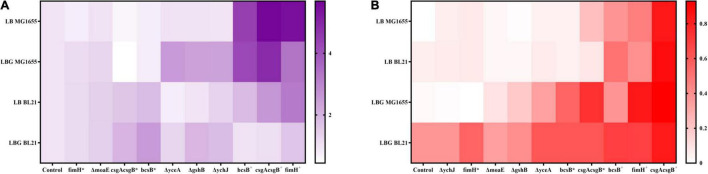
A summary and heat map illustration of **(A)** fold changes in biofilm biomass and **(B)** CR adsorption for different *E. coli* recombinant strains. Data is processed as described in Supplementary Material.

Disruption of *moaE*, *gshB*, *yceA*, or *ychJ* gene did not show significant effect on biofilm formation in LB medium. However, when grown in LBG medium the *gshB*-, *yceA*-, and *ychJ*-disrupted strains of both BL21 and MG1655 showed greatly enhanced biofilm formation (Figure 2). Among them, the *yceA*-disrupted strains also showed enhanced adsorption of CR (Figure 3). The overall results are summarized in Figure 4 which shows that most of genetic manipulations could promote biofilm formation to some extent.

### Effects of Biofilm Related Genes on Human Epidermal Growth Factor Secretion

**FIGURE 5:**
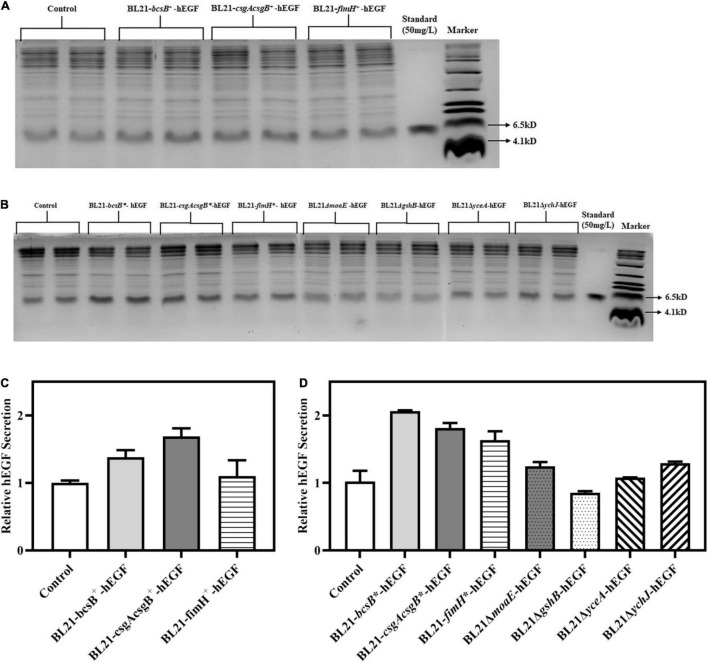
Effects of different gene manipulations on hEGF secretion in free-cell fermentation in LB medium. **(A)** SDS-PAGE of secreted hEGF in strains with plasmid-based expression of biofilm related genes. **(B)** SDS-PAGE of secreted hEGF in strains with genome-integrated expression of or disruption of biofilm related genes. **(C)** Relative hEGF secretion of strains with plasmid-based expression of biofilm related genes. **(D)** Relative hEGF secretion of strains with genome-integrated expression of or disruption of biofilm related genes. The hEGF secretion levels were normalized to the OD_600_ of corresponding culture.

Despite the genes studied above having some good effects on biofilm formation, their impacts on hEGF secretion needed to be investigated. So, the pET30a-hEGF plasmid was introduced into different BL21 recombinant strains, and hEGF secretion by these strains in LB medium was determined and normalized to the OD_600_ of corresponding culture (Supplementary Table 2). Results showed that compared with the hEGF secretion by wild BL21 strain (around 24 mg/L), overexpression of *bcsB*, *csgAcsgB*, and *fimH* by plasmid increased hEGF secretion by 38, 69, and 10%, respectively. By contrast, genome-integrated expression of these three genes could improve hEGF secretion to a more extent, showing an improvement by 62 to 105%, respectively (Figure 5). Genome-integrated expression of these genes was better for cell growth compared to plasmid-based expression, which might help hEGF secretion.

Although disruption of *moaE* and *ychJ* in *E. coli* had shown no apparent effects on biofilm formation in LB medium, it resulted in an improvement in hEGF secretion by 32 and 28%, respectively (Figure 5). This indicated that biofilm formation was not necessarily correlated with hEGF secretion, which meant that these gene disruptions might contribute to hEGF secretion by impacting on other aspects of cell physiology (e.g., the Sec machinery) rather than on biofilm formation. By contrast, disruption of *gshB* and *yceA* failed to improve hEGF secretion. Since LBG medium was previously shown to favor biofilm formation under some circumstances, it was also tried. Nevertheless, hEGF secretion would be severely reduced in LBG medium (data not shown).

### Biofilm-Based Fermentation for Continuous Human Epidermal Growth Factor Production

**FIGURE 6:**
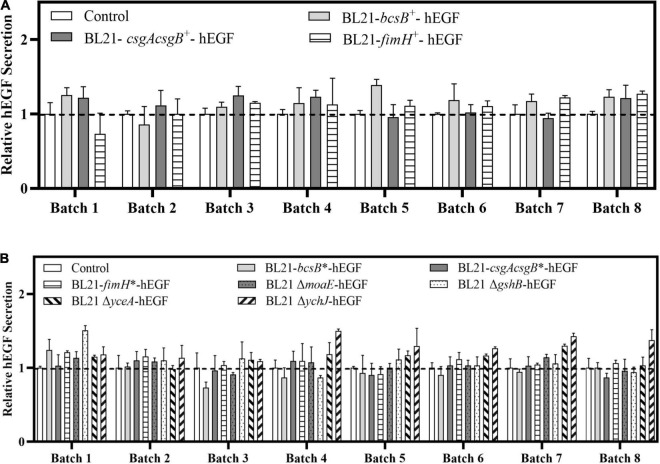
Effects of different gene manipulations on hEGF secretion in biofilm-based fermentation (repeated batch fermentation) in LB medium. **(A)** Relative hEGF secretion of strains with plasmid-based expression of biofilm related genes. **(B)** Relative hEGF secretion of strains with genome-integrated expression of or disruption of biofilm related genes.

Since cells can be immobilized in biofilm and can be used repeatedly, the feasibility of continuous production of hEGF through biofilm-based repeated-batch fermentation was explored, by providing cotton fabric in LB medium as biofilm carrier. For the plasmid-based expression strains BL21-*bcsB*^+^, BL21-*csgAcsgB*^+^, and BL21-*fimH*^+^, eight repeated batches were carried out. Results showed that the BL21-*bcsB*^+^ gave the best performance (Figure 6A). The hEGF production from seven out of the eight batches by BL21-*bcsB*^+^ was better than control, showing an overall average of 17% higher hEGF secretion (Table 2), while the production of BL21-*csgAcsgB*^+^ and BL21-*fimH*^+^ was also improved in general but seemed less effective. For the integrated expression strains and gene disrupted strains, eight repeated batches were carried out. It seemed that integration of *bcsB* reduced its positive effect on hEGF secretion observed previously. Integrated expression of *csgAcsgB* did not apparently improve hEGF secretion, while integrated expression of *fimH* showed apparent but somewhat unstable effects on hEGF production. For the gene disrupted strains except the *moaE*-disrupted strain, the hEGF secretion could all be improved (Figure 6B). The *yceA*-disrupted strain produced higher hEGF levels from seven of eight batches, showing an overall average of 14% higher hEGF production (Table 2). The best results were observed for the *ychJ*-disrupted strain whose hEGF production was higher than the BL21(DE3) wild strain during all the eight repeated batches, with an average of 28% higher hEGF production, increased from an average of 24 to 32 mg/L.

**TABLE 2:** Relative hEGF production (fold change) by recombinant *E. coli* strains with different genetic manipulations.

		Biofilm-based fermentation								
Genetic manipulation	Free-cell fermentation	Batch 1	Batch 2	Batch 3	Batch 4	Batch 5	Batch 6	Batch 7	Batch 8	Average
*bcsB* ^+^	1.38	1.25	0.86	1.10	1.15	1.39	1.19	1.17	1.23	1.17
*csgAcsgB* ^+^	1.69	1.21	1.11	1.25	1.23	0.96	1.02	0.94	1.21	1.12
*fimH* ^+^	1.10	0.73	1.00	1.15	1.13	1.11	1.10	1.22	1.27	1.09
bcsB*	2.06	1.24	1.02	0.73	0.87	0.93	0.90	0.94	1.00	0.95
csgAcsgB*	1.81	1.03	1.10	0.96	1.09	0.90	1.03	1.04	0.87	1.00
fimH*	1.63	1.21	1.15	1.04	1.09	0.92	1.11	0.95	1.06	1.07
Δ*moaE*	1.25	1.13	1.08	0.91	1.07	1.00	1.03	1.14	0.96	1.04
Δ*gshB*	0.85	1.51	1.10	1.13	0.87	1.11	1.03	1.06	0.94	1.09
Δ*yceA*	1.08	1.15	0.98	1.11	1.19	1.17	1.16	1.30	1.03	1.14
Δ*ychJ*	1.29	1.18	1.14	1.09	1.50	1.30	1.26	1.43	1.37	1.28

**FIGURE 7:**
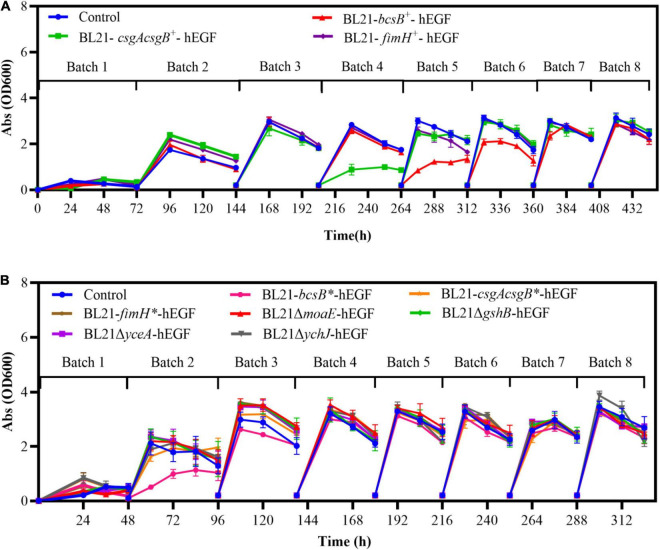
Cell densities (OD_600_) in the liquid cultures during biofilm-based repeated batch fermentation. **(A)** Strains with plasmid-based expression of biofilm related genes. **(B)** Strains with genome-integrated expression of or disruption of biofilm related genes.

Besides the higher hEGF production, cell growth and fermentation rate of the biofilm cells could also be improved. When *bcsB*, *csgAcsgB*, and *fimH* were expressed on a plasmid, the cell growth was slowed down and a time of 72 h was required for free-cell fermentation. By contrast, the time required for the biofilm-based fermentation was decreased gradually with increasing batches, and the final fermentation time was only 42 h. For the integrated expression strains and gene disrupted strains whose growth was generally better, the biofilm-based fermentation time was shortened to 36 h, compared to a fermentation time of 48 h for free-cell fermentation. Different from free cells, the biofilm retained cells so that they could be used repeatedly. The existing cells retained in the biofilm at the start of each new batch and their already established production capacity would contribute to the accelerated fermentation rate. In addition, biofilm cells attached on carriers could keep growing throughout the fermentation and reach a cell density much higher than that in liquid culture. This could increase the overall cell density and thus contribute to the accelerated fermentation rate also. Indeed, the maximum cell densities (OD_600_) of all the recombinant strains during free-cell fermentation were 1.5–2.5, whereas the cell densities during biofilm-based fermentation were all increased over 3.0 in the liquid culture. The improved cell growth during biofilm fermentation compensated for growth defects of some recombinant strains during traditional free-cell fermentation. For example, when the *gshB*-disrupted strain was applied to free-cell fermentation, its growth rate was significantly reduced with an OD_600_ of 1.7 which was 43% lower than that of BL21-hEGF, and the hEGF production was not improved. However, when it was applied to biofilm-based fermentation, the cell density was recovered to normal range (Figure 7) and the hEGF production was 9% higher than that of BL21-hEGF instead.

Interestingly, during the biofilm-based repeated batch fermentation, the cell densities in the liquid cultures of all the strains were very low in the first batch, consistent with an observation of clear fermentation broth. The cell densities of the liquid cultures were increased through the second batch, and most of them reached the maximum level in the third batch and remained stable hereafter (Figure 7). This indicated that cells were effectively adsorbed on the carrier at the early fermentation stages, and when enough cells were on the carrier they could be detached reversibly from the biofilm. However, the cell density profiles of the *bcsB* and *csgAcsgB* overexpression strains during the biofilm-based fermentation were somewhat different, that is, the cell densities in the liquid cultures were observed to drop significantly in the fourth and fifth batches (Figure 7A). Similarly, the cell density of the *bcsB* integrated expression strain during the second and third batches of biofilm fermentation was also obviously lower (Figure 7B). This probably suggested that expression of *bcsB* and *csgAcsgB* genes effectively increased the extracellular matrix synthesis which made cells better adsorbed to the carrier.

Biofilm development is a complex process that can alter growth, metabolism, and physiological properties of the cells. So, the performance of biofilm-based fermentation by some strains differed from that of free-cell fermentation. Although the *csgAcsgB* integrated expression strain and *moaE*-disrupted strain apparently promoted hEGF production compared to BL21(DE3) in free-cell fermentation, their performances became worse in biofilm-based fermentation. Overall, disruption of *ychJ* gave the best result, followed by overexpression of *bcsB*. These two recombinant strains apparently improved hEGF production in both free-cell fermentation and biofilm-based fermentation. The *ychJ* gene encodes a protein of yet unknown function. YchJ homologs were found in many other bacterial species but were not characterized either. Bioinformatical analysis showed that YchJ contains SecA-like metal-binding domains (MBD) that could coordinate zinc or iron and bind to SecB and ribosomes (Cranford-Smith et al., 2020). This suggested that YchJ might potentially interact with the Sec protein translocation machinery, possibly by competing with SecA, and thus its disruption increased hEGF secretion. However, this hypothesis needs to be further investigated. Overexpression of *bcsB* by plasmid showed great potential in continuous immobilized fermentation as the biofilm cells could be better immobilized. In the present study, genes were disrupted or overexpressed individually. Combined disruption or expression for multiple genes will be carried out to evaluate possible synergistic effects.

## Conclusion

Different from common strategies employed to improve the production efficiency of heterogeneous proteins in *E. coli*, this study employed biofilm-based systems to develop efficient hEGF production processes. Both plasmid-based and genome-integrated expression of *bcsB*, *fimH*, and *csgAcsgB*, and disruption of *moaE*, *gshB*, *yceA*, and *ychJ* were examined for their effects on biofilm formation and hEGF secretion. The best result was obtained from the *ychJ*-disrupted strain, which produced an average of 28% higher hEGF over the BL21(DE3) wild strain in the biofilm-based continuous fermentation. Overexpression of *bcsB* by plasmid also showed great potential in immobilized fermentation as the biofilm cells could be better immobilized. These genes could potentially be engineering targets for the design of cell factories with improved protein secretory capacity in future.

## Data Availability Statement

The original contributions presented in the study are included in the article/Supplementary Material, further inquiries can be directed to the corresponding author.

## Author Contributions

DL conceived and designed the experiments. ML and ZheW performed the laboratory work, analyzed and interpreted the data, and drafted the manuscript. MZ and CZ constructed the plasmids and strains. KZ and SL participated in the fermentation experiments. JL, ZhiW, and XS contributed to the experimental design, data interpretation, and critically revised the manuscript. All authors contributed to the article and approved the submitted version.

## Conflict of Interest

The authors declare that the research was conducted in the absence of any commercial or financial relationships that could be construed as a potential conflict of interest.

## Publisher’s Note

All claims expressed in this article are solely those of the authors and do not necessarily represent those of their affiliated organizations, or those of the publisher, the editors and the reviewers. Any product that may be evaluated in this article, or claim that may be made by its manufacturer, is not guaranteed or endorsed by the publisher.
