# Supplementary material for: Continuous Production of Human Epidermal Growth Factor Using Escherichia coli Biofilm
Source: Front Microbiol. 2022 Apr 12;13:855059. doi: 10.3389/fmicb.2022.855059 (PMC9039743; doi:10.3389/fmicb.2022.855059)
Supplement: Supplementary file 1 [file Data_Sheet_1.docx]

Supplementary Material

1. **Mutant Strains Construction**

For plasmid-based expression, the genes were individually ligated to the expression plasmid pBbE1a (with restriction enzyme BglII and AvrII) by using the ClonExpress II One Step Cloning Kit C112-01 (Vazyme, Nanjing, China). The biofilm genes *bcsB*, *csgAcsgB* and *fimH* were amplified from the genomic DNA of *E. coli* MG1655, and a terminator was amplified from the vector pBbE1a, using premiers NO.1 to 10 in Table S1. For genome-integrated expression, the *bcsB*, *csgAcsgB* and *fimH* were inserted in the *moaE* site by CRISPR. Vector pBbE1a was used as template to obtain the *trc* promoter by PCR. Each target gene fragment and upstream and downstream homologous sequences of *moaE* site were obtained by PCR using *E. coli* genome as templates. Primers used to obtain these fragments are listed in Table S1 (No.11 to 40). These fragments were ligated by overlap PCR. For gene disruption by CRISPR, primers NO.41 to 56 listed in Table S1 were used to amplify upstream and downstream homologous fragments for the target genes, using *E. coli* BL21(DE3) genome as template. These two fragments were ligated by overlap PCR. The N20 sequence of the sgRNA in pTarget was re-designed to target specific gene locus, using primers No.57 to 64 in Table S1. Mutant strains were verified for the correct genotype using checking primers No.65 to 72 listed in Table S1.

# Heat map illustration of fold changes in biofilm biomass

The fold changes in biofilm biomass of different recombinant strains, relative to that of the corresponding control (either BL21(DE3) wild strain, or the MG1655 wild strain), were calculated and used to draw the heat map in Figure 4A. The biofilm biomass was measured as the absorbance at 570 nm (OD_570_) of crystal violet extracted from biofilm in each well of a 96-well plate. The fold change was calculated as OD_570(recombinant)_/OD_570(Control)_. The relative biofilm biomass of control was set as 1.0.

For CR adsorption, the residual amount of CR in the supernatant was measured as absorbance at 485 nm (OD_485_). The ratio of CR adsorption was calculated by the following formula: 1-OD_485_/OD_485(PBS+CR)_, wherein OD_485(PBS+CR)_ is the absorbance at 485 nm of CR dissolved in PBS.

# Solutions required for SDS-PAGE

For SDS-PAGE, the gels were running in 1× SDS-PAGE running buffer at a voltage of 80 V for 60 min and then at 100 V for 80 min. Solutions required in this study are listed as follows:

4× SDS-PAGE running buffer (containing Tris 12 g/L and glycine 57.6 g/L), 10% SDS.

Staining solution (containing methanol 225 mL, glacial acetic acid 50 mL, pure water 225 mL, Coomassie brilliant blue 2.5 g).

Decolorizing solution (containing glacial acetic acid 100 mL, ethanol absolute 50 mL, pure water 850 mL).

**TABLE S1** Primers used in this study.

| Primer | Sequence Number | Sequence |
| --- | --- | --- |
| pBbE1a-*bcsB*-F | NO.1 | TAACAATTTCAGAATTCAAAAGATCTTTTAAGAAGGAGATATACATATGAAAAGAAAACTATTCTG |
| pBbE1a-*bcsB*-R | NO.2 | CTTTCGTTTTATTTGATGCCTGGTTACTCGTTATCCGGGTTAAGAC |
| pBbE1a-*bcsB*-terminator-F | NO.3 | GTCTTAACCCGGATAACGAGTAACCAGGCATCAAATAAAACGAAAG |
| pBbE1a-terminator-F | NO.4 | CGCTCGCCGCAGCCGAACGCCCTAGGTATAAACGCAGAAAGGCCCA |
| pBbE1a-*csg*-F | NO.5 | TAACAATTTCAGAATTCAAAAGATCTTTTAAGAAGGAGATATACATATGAAAAACAAATTGTTATT |
| pBbE1a-*csg*-R | NO.6 | CTTTCGTTTTATTTGATGCCTGGTTAGTACTGATGAGCGGTCG |
| pBbE1a-*csg*-terminator-F | NO.7 | ACGCGACCGCTCATCAGTACTAACCAGGCATCAAATAAAACGAAAG |
| pBbE1a-*fimH*-F | NO.8 | TAACAATTTCAGAATTCAAAAGATCTTTTAAGAAGGAGATATACATATGAAACGAGTTATTACCCT |
| pBbE1a-*fimH*-R | NO.9 | CTTTCGTTTTATTTGATGCCTGGTTATTGATAAACAAAAGTCACGC |
| pBbE1a-*fimH*-terminator-F | NO.10 | GCGTGACTTTTGTTTATCAATAACCAGGCATCAAATAAAACGAAAG |
| *bcsB* pTrc-F | NO.11 | CTTATCATCGACTGCACGGT |
| *bcsB* pTrc-R | NO.12 | CAGAATAGTTTTCTTTTCATATGTATATCTCCTTCTTAAAAG |
| *bcsB*-F | NO.13 | TAAGAAGGAGATATACATATGAAAAGAAAACTATTCTGGA |
| *bcsB*-R | NO.14 | ATTCATCTTCAATTACTCGTTAT |
| *bcsB* up-F | NO.15 | GCTCAGCAAAGTTGAAGTCAAT |
| *bcsB* up-R | NO.16 | ACCGTGCAGTCGATGATAAGAATTTTGGTTTCTGCCATCTTAACCT |
| *bcsB* Insert-F | NO.17 | AGATGGCAGAAACCAAAATTCTTATCATCGACTGCACGGT |
| *bcsB* Insert-R | NO.18 | AACAAAAAACTACCAGCGTTATTCATCTTCAATTACTCGTTATCCG |
| *bcsB* down-F | NO.19 | ACGAGTAATTGAAGATGAATAACGCTGGTAGTTTTTTGTT |
| *bcsB* down-R | NO.20 | TAACGACGAAAGTACTGGCGAT |
| *csg* pTrc-F | NO.21 | AGATGGCAGAAACCAAAATTCGACTGCACGGTGCA |
| *csg* pTrc-R | NO.22 | ACATCATAAATAACAATTTGTTTTTCATTGAAATTGTTATCCGCTCACAAT |
| *csg*-F | NO.23 | TTGTGAGCGGATAACAATTTCAATGAAAAACAAATTGTTATTTATGATGT |
| *csg*-R | NO.24 | AACAAAAAACTACCAGCGTTTTAGTACTGATGAGCGGTCG |
| *csg* up-F | NO.25 | GCTCAGCAAAGTTGAAGTCAAT |
| *csg* up-R | NO.26 | TGCACCGTGCAGTCGAATTTTGGTTTCTGCCATCTTAACC |
| *csg* Insert-F | NO.27 | AGATGGCAGAAACCAAAATTCGACTGCACGGTGCA |
| *csg* Insert-R | NO.28 | AACAAAAAACTACCAGCGTTTTAGTACTGATGAGCGGTCG |
| *csg* down-F | NO.29 | CGACCGCTCATCAGTACTAAAACGCTGGTAGTTTTTTGTT |
| *csg* down-R | NO.30 | TAACGACGAAAGTACTGGCGAT |
| *fimH* pTrc-F | NO.31 | AGATGGCAGAAACCAAAATTCGACTGCACGGTGCA |
| *fimH* pTrc-R | NO.32 | CAGCAAACAGGGTAATAACTCGTTTCATTGAAATTGTTATCCGCTCACAAT |
| *fimH*-F | NO.33 | TTGTGAGCGGATAACAATTTCAATGAAACGAGTTATTACCCTGT |
| *fimH*-R | NO.34 | AACAAAAAACTACCAGCGTTTTATTGATAAACAAAAGTCACGCCAAT |
| *fimH* up-F | NO.35 | GCTCAGCAAAGTTGAAGTCAAT |
| *fimH* up-R | NO.36 | TGCACCGTGCAGTCGAATTTTGGTTTCTGCCATCTTAACC |
| *fimH* Insert-F | NO.37 | AGATGGCAGAAACCAAAATTCGACTGCACGGTGCA |
| *fimH* Insert-R | NO.38 | AACAAAAAACTACCAGCGTTTTATTGATAAACAAAAGTCACGCCAAT |
| *fimH* down-F | NO.39 | TGACTTTTGTTTATCAATAAAACGCTGGTAGTTTTTTGTT |
| *fimH* down-R | NO.40 | TAACGACGAAAGTACTGGCGAT |
| *moaE* up-F | NO.41 | GGATCTGATCCCGCTCTGTCAT |
| *moaE* up-R | NO.42 | AAAAACTACCAGCGTTAATTTTGGTTTCTGCCATCTTAACCTCC |
| *moaE* down-F | NO.43 | GCAGAAACCAAAATTAACGCTGGTAGTTTTTTGTTAGCCG |
| *moaE* down-R | NO.44 | GTAACGACGAAAGTACTGGCGA |
| *gshB* up-F | NO.45 | TCTCCGCTGCATATTCACCT |
| *gshB* up-R | NO.46 | TTAACATTCCGATGCCGAGCTTGATCATTA |
| *gshB* down-F | NO.47 | GCTCGGCATCGGAATGTTAATGGATGCCAT |
| *gshB* down-R | NO.48 | TACGTCAGACGGTTGTTTATCG |
| *yceA* up-F | NO.49 | CATGCCAACGGATTCGAAAT |
| *yceA* up-R | NO.50 | GGCAATGATATTTATTGTGTAACACTGGCATGG |
| *yceA* down-F | NO.51 | CCAGTGTTACACAATAAATATCATTGCCGGATGC |
| *yceA* down-R | NO.52 | GTCAGCACGCCTTTGTTAAT |
| *ychJ* up-F | NO.53 | TCGCCTTCAGTAAACCATAATTC |
| *ychJ* up-R | NO.54 | TTTTAAATTTTTTACACAAAGCTGAGACACAAATAAT |
| *ychJ* down-F | NO.55 | GTGTCTCAGCTTTGTGTAAAAAATTTAAAAAGTGCTGCGG |
| *ychJ* down-R | NO.56 | AGTCAGGTTGATAAGCATCAATG |
| *moaE* sg-F | NO.57 | CATCCCCGGATAGTGTTCGAGTTTTAGAGCTAGAAATAGCAAGTTAAAATAAGGCTAGT |
| *moaE* sg-R | NO.58 | TCGAACACTATCCGGGGATGACTAGTATTATACCTAGGACTGAGCTAGCTGT |
| *gshB* sg-F | NO.59 | CGTTAAGTCAGAGAACCAGGGTTTTAGAGCTAGAAATAGCAAGTTAAAAT |
| *gshB* sg-R | NO.60 | CCTGGTTCTCTGACTTAACGACTAGTATTATACCTAGGACTGAGCT |
| *yceA* sg-F | NO.61 | TCGCCCAAAAACATTCAGCGGTTTTAGAGCTAGAAATAGCAAGTTAAAAT |
| *yceA* sg-R | NO.62 | CGCTGAATGTTTTTGGGCGAACTAGTATTATACCTAGGACTGAGCT |
| *ychJ* sg-F | NO.63 | GCATCCCTCTTGTGGAGCAGGTTTTAGAGCTAGAAATAGCAAGTTAAAAT |
| *ychJ* sg-R | NO.64 | CTGCTCCACAAGAGGGATGCACTAGTATTATACCTAGGACTGAGCT |
| *moaE* check-F | NO.65 | GTATGTCGCAACTGACCCATAT |
| *moaE* check-R | NO.66 | CCATATTTTTCAGTTTCTGCGT |
| *gshB* check-F | NO.67 | AGGTCTTTGACGCCGAAATT |
| *gshB* check-R | NO.68 | CGGCGTTTTGAACAGAATAT |
| *yceA* check-F | NO.69 | GAATGTGTTCCATGCCGATC |
| *yceA* check-R | NO.70 | AAGCCTGCTTGGTTTAACCT |
| *ychJ* check-F | NO.71 | TGAGGGAAATAGGAATTGGGTT |
| *ychJ* check-R | NO.72 | CAATAAACGCTGGCAGGAATGA |

**TABLE S2.** Cell density (OD_600_) of BL21(DE3) recombinants in free-cell hEGF fermentation at 48 h.

| Genetic manipulation | OD_600_-R1 | OD_600_-R2 |
| --- | --- | --- |
| Control 1^a^ | 1.606 | 1.588 |
| *bcsB*^+^ | 1.654 | 1.668 |
| *csgAcsgB*^+^ | 1.346 | 1.313 |
| *fimH*^+^ | 2.338 | 1.758 |
| Control 2^b^ | 2.415 | 2.457 |
| *bcsB** | 1.930 | 1.848 |
| *csgAcsgB** | 1.954 | 1.866 |
| *fimH** | 1.956 | 1.930 |
| Δ*moaE* | 2.111 | 2.152 |
| Δ*gshB* | 1.690 | 1.698 |
| Δ*yceA* | 2.313 | 2.210 |
| Δ*ychJ* | 2.118 | 2.179 |

^a^Control 1, BL21(DE3) contained empty pBbE1a plasmid as well as pET30a-hEGF;

^b^Control 2, BL21(DE3) contained only pET30a-hEGF.

R1 and R2 represent two replicates.
